# Assessment of retinal vascular network in amnestic mild cognitive impairment by optical coherence tomography angiography

**DOI:** 10.1371/journal.pone.0233975

**Published:** 2020-06-03

**Authors:** Chiara Criscuolo, Gilda Cennamo, Daniela Montorio, Antonio Carotenuto, Alfonso Strianese, Elena Salvatore, Fausto Tranfa, Giovanni Cennamo, Roberta Lanzillo, Vincenzo Brescia Morra

**Affiliations:** 1 Department of Neurosciences, Reproductive and Odontostomatological Sciences, “Federico II” University Naples, Naples, Italy; 2 Public Health Department, Eye Clinic, University of Naples Federico II, Naples, Italy; Weill Cornell Medicine-Qatar, QATAR

## Abstract

**Objective:**

To assess the presence of retinal vascular network abnormalities in amnestic mild cognitive impairment (aMCI) patients and healthy subjects (HS) through optical coherence tomography angiography (OCTA).

**Methods:**

OCTA and SD-OCT were performed in aMCI patients and cognitive normal HS. A complete neuropsychological evaluation was performed. Differences in vessel density (VD) in each retinal vascular plexus and in foveal avascular zone (FAZ) were evaluated with linear mixed model after correction for age, sex and disease duration.

**Results:**

Twenty-seven aMCI patients (10 Single domain aMCI, 17 Multidomain aMCI) and 29 HS were enrolled. aMCI patients showed a statistically significant reduced VD in superficial capillary plexus (SCP), deep capillary plexus (DCP) and an increased FAZ compared to controls. When aMCI patients were divided in single domain (SD) and multiple domains (MD) aMCI, SD aMCI showed no VD differences in SCP, DCP and Radial Peripapillary Capillary, while the FAZ area was significantly larger compared to controls. In MD aMCI, VD values were lower and FAZ was increased compared to controls. Comparing both aMCI groups, MD aMCI showed a significant reduction in VD values of SCP. No correlation was found between mini mental state examination (MMSE) scores and OCTA parameters.

**Conclusions:**

OCTA is able to detect changes in retinal microvascular network in early cognitive deficits and, the most sensitive alteration seems to be the enlargement of the FAZ. This non-invasive tool provides useful information on retinal involvement patterns in MCI diagnosis and follow up. Vascular network impairment seems to be related to the number of domains affected and not to MMSE.

## Introduction

Mild cognitive impairment (MCI) is a cognitive decline more accentuated than expected considering the age of the patient, but not enough to compromise daily life activities [1]. Currently, the diagnosis of MCI relies purely on clinical evaluation, including neuropsychological testing [2]. Individuals with a clinical diagnosis of the amnestic type of MCI (aMCI) represent a high-risk target population for progression to Alzheimer’s disease (AD). The rate of conversion to AD is 1% to 2% per year in the general population, 5% to 10% in subjects with MCI [3], and up to 50% in 30 months for aMCI [4]. Yet, many patients with MCI remain stable and do not develop dementia. Although the mechanisms responsible for the onset and progression of MCI have been subject of many studies, questions remain, especially about the transition from MCI to AD and factors predicting conversion.

In this scenario, the development of new biomarkers for the diagnosis and follow-up of patients with MCI is crucial. Optical coherence tomography angiography (OCTA) is non-invasive diagnostic tool that can acquire high-resolution, in vivo cross-sectional images, quantitative and reproducible measurements of macular and peripapillary vascular networks [4,5].

Previous studies, using OCTA, identified the reduction in retinal vascular flow in MCI and AD patients compared to controls suggesting a vascular role in the pathophysiology of these diseases [6–10]. Extensive evidences showed that the vascular factors contribute to cerebral neurodegeneration in AD [11].

In particular vascular alterations have been reported in AD including impairment of blood–brain barrier, decreased vascular density, vascular diameter and blood flow [12] that may indicate the presence of a vasculopathy in the pathogenesis of AD [13]. Global and focal cerebral hypoperfusion measured by transcranial Doppler, single-photon emission computed tomography, and arterial spin MRI is not only evident in AD, but also in MCI [14,15].

Similar to the brain, the retina has a highly isolated and protected vascular system and shares similar physiological and anatomical features with the brain [12,16]. Therefore, the goal of our study was to analyze the retinal vascular network through OCTA in healthy controls and patients with aMCI, in order to verify the presence of vascular abnormalities in aMCI and evaluate the correlation between OCTA parameters and the disease stage.

## Methods

### Study population

MCI patients were recruited consecutively from the Dementia out-clinic of “Federico II” University of Naples Neurology Department, from January to May 2019.

MCI participants initially were evaluated and diagnosed clinically by experienced neurologists with a specialization in memory disorders (C.C. and E.S.) based on the clinical guidelines and recommendations of the National Institute on Aging-Alzheimer’s Association [17]. Clinical history, cognitive testing, and neuroimaging were reviewed for diagnostic accuracy by a group consensus conference that included neurologists, psychiatrists, and neuropsychologists to discussed and confirm the diagnosis of MCI.

All aMCI diagnoses required a detailed research clinical assessment to rule out additional medical causes and a formal neuropsychological battery as described in the Neuropsychological Assessment section. In addition to specific neurocognitive performance metrics, the diagnosis of MCI required preservation of activities of daily living. Thus, all participants had a study partner who completed the Activities of Daily Living Questionnaire [17]. All patients performed brain MRI.

Whenever possible, available biomarker data were also included in consideration of the final diagnosis [18]. Apolipoprotein E e4 (apoE4) was performed in four patients, PET imaging in ten. None performed lumbar puncture.

Inclusion criteria were: normal ophthalmic examination, no history of intraocular surgery or retinal pathological features.

Exclusion criteria included the presence of congenital eye disorders, myopia greater than 6 diopters, history of ocular surgery, presence of significant lens opacities or any macular disease, previous diagnosis of glaucoma, evidence of vitreoretinal disease, uveitis and diabetic retinopathy, history of other neurological or psychiatric disorders and low-quality images obtained with OCT.

Patients with a history of stroke, coagulopathy, diabetes, uncontrolled hypertension, head trauma, alcohol or drug addiction, or depression were also excluded.

Patients with aMCI were classified into two subtypes, a single domain (SD) aMCI group and a multiple domain (MD) aMCI group. SD aMCI subjects scored outside the range of normality in at least one of the memory tests and performed within the normality cut-off scores in tests exploring all other cognitive domains.

MD aMCI showed at least another compromised domain beyond memory (see below for detailed description of the neuropsychological testing). Patients with non-amnestic MCI were excluded.

Cognitively normal (CN) controls were enrolled during the same study period from subjects who received annual eye examinations or from the family members of the patients (spouses). The control group was constituted by CN healthy subjects similar, in age and sex, who fit the same inclusion and exclusion criteria upon reported. None complained of cognitive problems or had any evidence of cognitive deficits on neuropsychological testing.

Evaluation of best-corrected visual acuity (BCVA) according to the Early Treatment of Diabetic Retinopathy Study (ETDRS), slit-lamp biomicroscopy, fundus examination with a +90 D lens, spectral domain (SD)-OCT and OCT angiography were performed in all patients and controls.

The study was approved by the Institutional Review Board of the University of Naples “Federico II” (protocol number: 142/19) and all investigations adhered to the tenets of the Declaration of Helsinki. Signed informed consents were obtained from each subject.

### Neuropsychological assessment

Cognitive functions were assessed in all subjects using an extensive neuropsychological battery. Testing was administered by two trained neuropsychologists. The battery included a general cognitive evaluation, using the Mini Mental State Evaluation [19], plus specific tests for several cognitive domains: (1) *Long*-*term memory*: Immediate and Delayed recall of a 15-Word List (I-Re and D-Re) [20], Short Story Recall (SSRe) [21], Delayed recall of Complex Rey's Figure [22]; (2) *Short*-*term memory*: Digit span and Corsi Block Tapping task [23]; (3) *Language*: Token Test [24,25]; (4) *Reasoning*: Raven's Coloured Progressive Matrices [21]; (5) *Executive functions and attention*: Phonological Word Fluency [21], Categorical Word Fluency [22], Attentional Matrices [22], *Praxis*: Copy of drawings [22,23], Copy of Complex Rey's Figure [23]. Adjustments for sex, age and education were applied according to Italian normative data.

### Spectral domain optical coherence tomography

The mean circumpapillary RNFL and GCC thickness were evaluated, after pupillary dilation (minimum diameter 5 mm), with SD-OCT (software RTVue XR version 2017.1.0.151, Optovue Inc., Fremont, CA, USA) which captures 26,000 axial scans (A-scans) per second and provides a 5-μm depth resolution in tissue. The optic nerve head map protocol evaluated the circumpapillary RNFL. This protocol automatically generated a circumpapillary RNFL thickness map based on measurements obtained around a circle 3.45 mm in diameter centred on the optic disc. The GCC scan was centred 1-mm temporal to the fovea and covered a square grid (7 mm × 7 mm) on the central macula. The GCC thickness was measured from the internal limiting membrane to the outer boundary of the inner plexiform layer.

Only high-quality images, as defined by a signal strength index above 40, were accepted. The device software generates a significance map with normative database comparison for GCC thickness [26].

The examiner rejected scans that had motion artefacts, poor centration, incorrect segmentation or poor focus.

### OCT angiography

The XR Avanti AngioVue OCTA (software ReVue version 2017.1.0.151, Optovue Inc., Fremont, CA, USA), is a device with a high speed of 70 000 axial scans per second that uses a light source of 840 nm and an axial resolution of 5 μm. This system is based on split-spectrum amplitude de-correlation algorithm (SSADA) which uses blood flow as intrinsic contrast. Flow is detected as a variation over time in a speckle pattern formed by the interference of light scattered by red blood cells and adjacent tissue structures [27].

The macular capillary network was visualized in scans centered on the fovea by performing a 6 x 6 mm scan. The OCT-A software, according to the ETDRS classification of diabetic retinopathy, analyzed the macular region divided in whole image, fovea and parafovea in each vascular network of the retina: Superficial Capillary Plexus (SCP) and Deep Capillary Plexus (DCP).

The superficial vascular plexus was selected at a 60 μm thickness from the inner limiting membrane to include all the vessels of this plexus. A 30 μm thick layer from the inner plexiform layer visualized the entire deep retinal plexus.

In each retinal vascular network, the software (AngioAnalytic^TM^) automatically calculated the vessel density (VD) that was defined as the percentage area occupied by vessels in the analyzed region on the OCTA en-face images [28].

The Angio Vue disc mode automatically segmented the VD of the Radial Peripapillary Capillary (RPC) analyzing the whole papillary region with an area scan of 4.5 x 4.5 mm. The VD, defined as the percentage of the peripapillary region occupied by blood vessels, was analyzed in the superficial retinal layers and extended from the Inner Limiting Membrane (ILM) to the RNFL posterior boundary [29].

The FAZ area was automatically calculated by the Angiovue software covering a 6 x 6 mm area over the macular region in the full retinal plexus and it was calculated in square millimetres [30].

From each analysis were excluded the images with a signal strength index less than 40, residual motion artefacts, incorrect segmentation, low centration and focus.

### Statistical analysis

Statistical analysis was performed with the Statistical Package for Social Sciences (Version 20.0 for Windows; SPSS Inc, Chicago, Ill, USA). For all variables, variance homogeneity and Gaussianity were tested with Shapiro-Wilk test. Two-tailed Student-t test was performed to assess differences in standard OCT measures between healthy controls and MCI patients. One-way analysis of variance (ANOVA) followed by Bonferroni post hoc analysis was used to evaluate differences in structural SD-OCT parameters between healthy controls, SD and MD MCI patients. Differences in OCTA parameters between groups were tested with linear mixed model with random slopes and intercepts using a Bonferroni correction for multiple comparisons, including age, sex and disease duration as covariates, group as factor of interest and VD in each retinal vascular plexus (SCP, DCP, RPC) and FAZ area as dependent variable. In order to assess contribute from both SD-OCT and OCTA measures on subject group, namely patients or healthy controls, we performed a linear mixed model with random slopes and intercepts using a Bonferroni correction for multiple comparisons, including group as dependent variable and VD in each retinal vascular plexus (SCP, DCP, RPC), FAZ area together with SD-OCT measures and age and sex as independent variables. We also assessed the correlations between SD-OCT parameters (Ganglion Cell complex (GCC) average, Retinal Nerve Fiber Layer (RNFL) average) and OCTA parameters (SCP, DCP, RPC and FAZ area) using a linear mixed model with age, sex as covariates in MCI patients. Subject ID was included in all models as random factor to account for within-subject inter-eye correlation. Finally, we performed the correlations between Mini Mental State Examination (MMSE) score and both OCT and OCTA parameters using a linear mixed model with age, sex and subject ID as covariates in MCI patients. As exploratory analysis, we also assessed correlations between OCTA parameters and scores for each cognitive test using a linear mixed model with age, sex and subject as covariates in MCI patients. A p value of < 0.05 (divided the number of multiple comparisons) was considered statistically significant.

## Results

We included fifty-four eyes from 27 patients with MCI and fifty-eight from 29 CN controls.

Ten patients had a SD aMCI (4 females, 6 males, mean age 71.3 ± 6 years), and 17 a MD aMCI (11 females, 6 males, 74 ± 6 years). Among MD aMCI patients, 13 had two impaired domains, two patients had three ones, one four and one five domains. Demographic and clinical features of included subjects are summarized in Table 1.

**Table 1:** Demographic and clinical characteristics of aMCI, SD aMCI, MD aMCI patients and cognitive normal controls.

	Control	aMCI	SD aMCI	MD aMCI
**Subjects (n.)**	29	27	10	17
**Age (years)**	73.1 ± 7	73 ± 6	71.3 ± 6	74 ± 6
**Sex (female/male)**	15/14	15/12	4/6	11/6
**MMSE score**	28.03 ± 1.3	26.51 ± 1.8	27 ± 2	26 ± 1.5
**Disease duration (months)**	-	19 ± 7	19 ± 4	20 ± 7
**OCTA parameters (%)**				
***SCP Whole***	48.12 ± 4.53	44.92 ± 5.04	47.84 ± 3.70	43.21 ± 4.97
***DCP Whole***	50.58 ± 4.69	45.13 ± 6.67	47.59 ± 6.70	43.69 ± 6.31
***RPC Whole***	48.82 ± 3	46.78 ± 4.44	48.74 ± 2.11	45.62 ± 5.05
***FAZ area (mm***^***2***^***)***	0.19 ± 0.06	0.28 ± 0.12	0.27 ± 0.12	0.28 ± 0.12
**SD-OCT parameters (μm)**				
***GCC average***	98.05 ± 6.11	91.72 ± 9.47	97.50 ± 10.83	88.21 ± 5.86
***RNFL average***	101.40 ± 9.14	96.43 ± 8.58	100.55 ± 9.24	94 ± 7.27
**BCVA (logMar)**	0.06 ± 0.08	0.05 ± 0.07	0.06 ± 0.08	0.04 ± 0.07

aMCI: Amnestic Mild Cognitive Impairment; SD: Single Domain; MD: Multiple Domain; MMSE: Mini Mental State Examination; OCTA: Optical Coherence Tomography Angiography; SCP: Superficial Capillary Plexus; DCP: Deep Capillary Plexus; RPC: Radial Peripapillary Capillary; FAZ: Fovea Avascular Zone; SD-OCT: Spectral Domain OCT; GCC: Ganglion Cell Complex; RNFL: Retinal Nerve Fiber Layers; BCVA: Best-corrected Visual Acuity; logMAR: Logarithm of the Minimum Angle of Resolution.

Patients and controls did not differ for age, sex and BCVA. Conversely, patients showed lower MMSE score.

When compared with controls, MCI patients had a lower GCC thickness (98.05 ± 6.11 vs 91.65 ± 9.16, p<0.001) and RNFL thickness (101.40 ± 9.14 vs 96.43 ± 8.58, p = 0.004) at SD-OCT and a reduced VD in SCP and DPC in whole macular region and an increased FAZ area at OCTA (β = -3.138, p = 0.007; β = -5.476, p<0.001; β = 0.085, p<0.001, respectively) after correction for age, gender and disease duration (Tables 2 and 3). When including both SD-OCT and OCTA measures in the same model we observed that MCI patients displayed an increased FAZ area (β = 18.92, p<0.001).

**Table 2:** Comparison in SD-OCT parameters among SD aMCI, MD aMCI patients and cognitively normal controls.

	Controls vs aMCI	Controls vs SD aMCI	Controls vs MD aMCI	SD aMCI vs MD aMCI	ANOVA
	P value*	**P value**^±^	**P value**^±^	**P value**^±^	**P value**^**±**^
**GCC average**	<0.001	1	<0.001	<0.001	<0.001
**RNFL average**	0.004	1	<0.001	0.025	<0.001

aMCI: Amnestic Mild Cognitive Impairment; SD: Single Domain; MD: Multiple Domain; GCC: Ganglion Cell Complex; RNFL: Retinal Nerve Fiber Layer; SD-OCT: Spectral Domain Optical Coherence Tomography

* Student t test, P value <0.05

^±^ ANOVA, followed by Bonferroni post hoc analysis. P value <0.05

**Table 3:** Comparison in OCTA parameters among SD aMCI, MD aMCI patients and cognitively normal controls.

	**Control vs aMCI**		
	**β**	**(95% CI)**	**P-value**
SCP Whole	-3.138	(-5.366 to -0.911)	0.007
DCP Whole	-5.476	(-7.846 to -3.105)	<0.001
RPC Whole	-2.063	(-4.084 to -0.041)	0.046
FAZ area	0.085	(0.043 to 0.128)	<0.001
**Control vs SD aMCI**			
** **	**β**	**(95% CI)**	**P-value**
SCP Whole	-0.486	(-3.386 to -2.414)	0.738
DCP Whole	-3.552	(-6.752 to -0.352)	0.030
RPC Whole	0.780	(-2.467 to -2.828)	0.892
FAZ area	0.082	(0.023 to 0.142)	0.007
	**Control vs MD aMCI**		
** **	**β**	**(95% CI)**	**P-value**
SCP Whole	-4.731	(-7.153 to -2.308)	<0.001
DCP Whole	-6.624	(-9.295 to -3.953)	<0.001
RPC Whole	-3.412	(-5.628 to -1.196)	0.003
FAZ area	0.087	(0.038 to 0.137)	0.001
	**SD aMCI vs MD aMCI**		
** **	**Β**	**(95% CI)**	**P-value**
SCP Whole	-4.245	(-7.423 to 1.066)	<0.001
DCP Whole	-3.072	(-6.583 to 0.439)	0.085
RPC Whole	-3.592	(-6.491 to -0.693)	0.016
FAZ area	0.005	(-0.060 to 0.070)	0.877

aMCI: Amnestic Mild Cognitive Impairment; SD: Single Domain; MD: Multiple Domain; OCTA: Optical Coherence Tomography Angiography; SCP: Superficial Capillary Plexus; DCP: Deep Capillary Plexus; RPC: Radial Peripapillary Capillary; FAZ: Foveal Avascular Zone; β: Regression Coefficient; CI: Confidence Interval; P<0.0125

**Fig 1:**
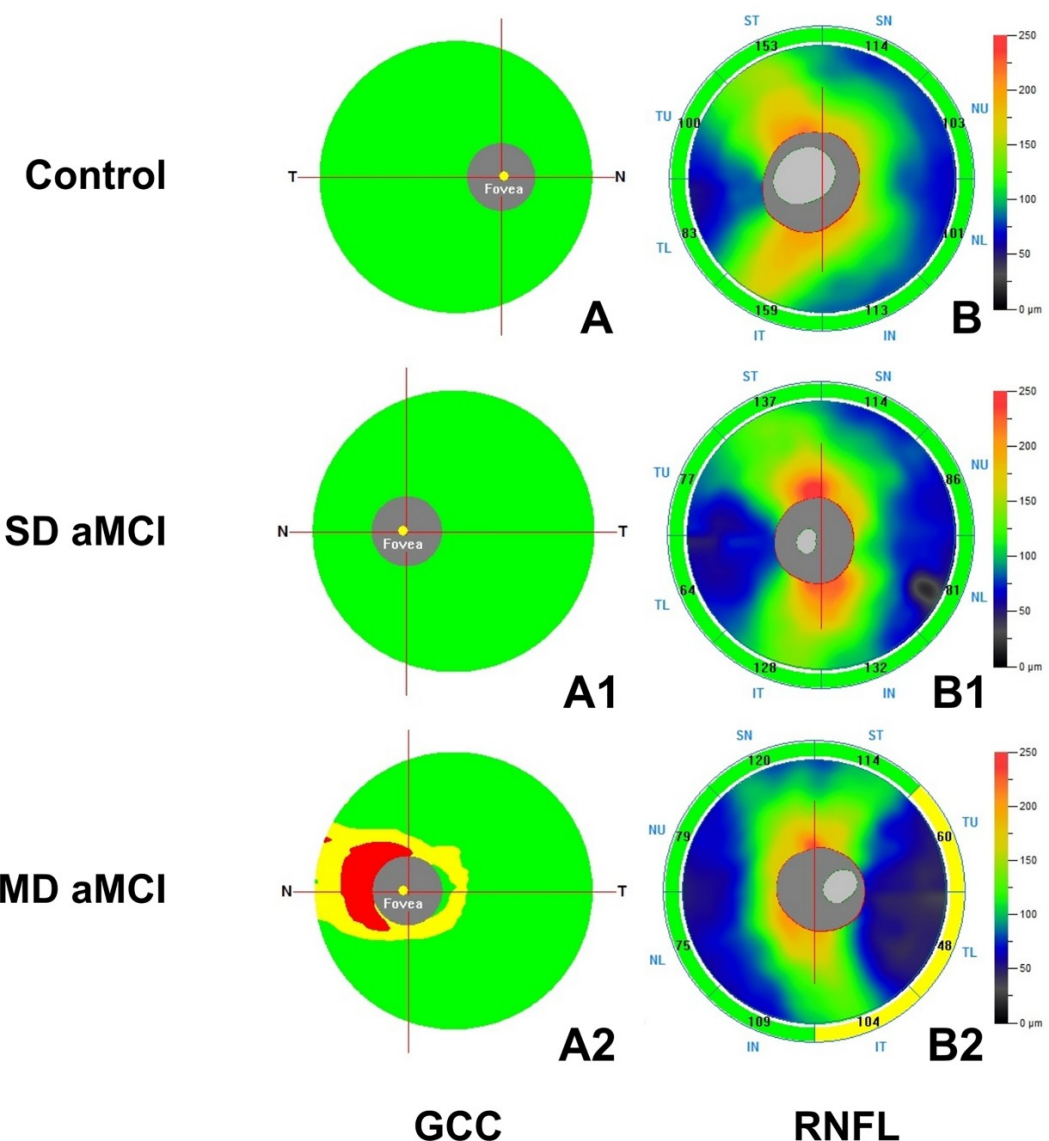
Optical coherence tomography (SD-OCT) images of the ganglion cell complex (GCC) and retinal nerve fiber layer (RNFL) in a control subject (male, 72 years) (A, B), in a patient with Single Domain amnestic Mild Cognitive Impairment (SD aMCI) (male, 70 years) (A1, B1) and in a patient with Multiple Domain aMCI (male, 73 years) (MD aMCI) (A2, B2). Compared with healthy control, SD aMCI patient showed no differences in GCC (A1) and RNFL (B1) while GCC (A2) and RNFL (B2) were significantly thinner in MD aMCI patient.

After dividing MCI group in SD and MD MCI patients, we reported a decrease in GCC only for MD MCI and not for SD MCI patients when compared with CN controls (MD MCI patients: 88.21 ± 5.86 vs CN controls: 98.05 ± 6.11, p<0.001, Table 2) (Fig 1). Similarly, we observed a reduced RNFL in MD MCI patients when compared with CN controls (MD MCI patients: 94 ± 7.27 vs CN controls: 101.40 ± 9.14, p<0.001, Table 2), whereas no difference in SD-OCT parameters was detected between CN controls and SD MCI patients (Fig 1).

The VD in all OCTA parameters did not differ between SD aMCI patients and controls except for FAZ area that turned out to be significantly larger than in controls (β = 0.085, p = 0.007). All OCTA parameters were significantly lower in MD aMCI patients respect to controls (SCP: β = -4.731, p<0.001; DCP: β = -6.624, p<0.001 and RPC: β = -3.412, p = 0.003, FAZ: β = 0.087, p = 0.001, Table 3).

**Fig 2:**
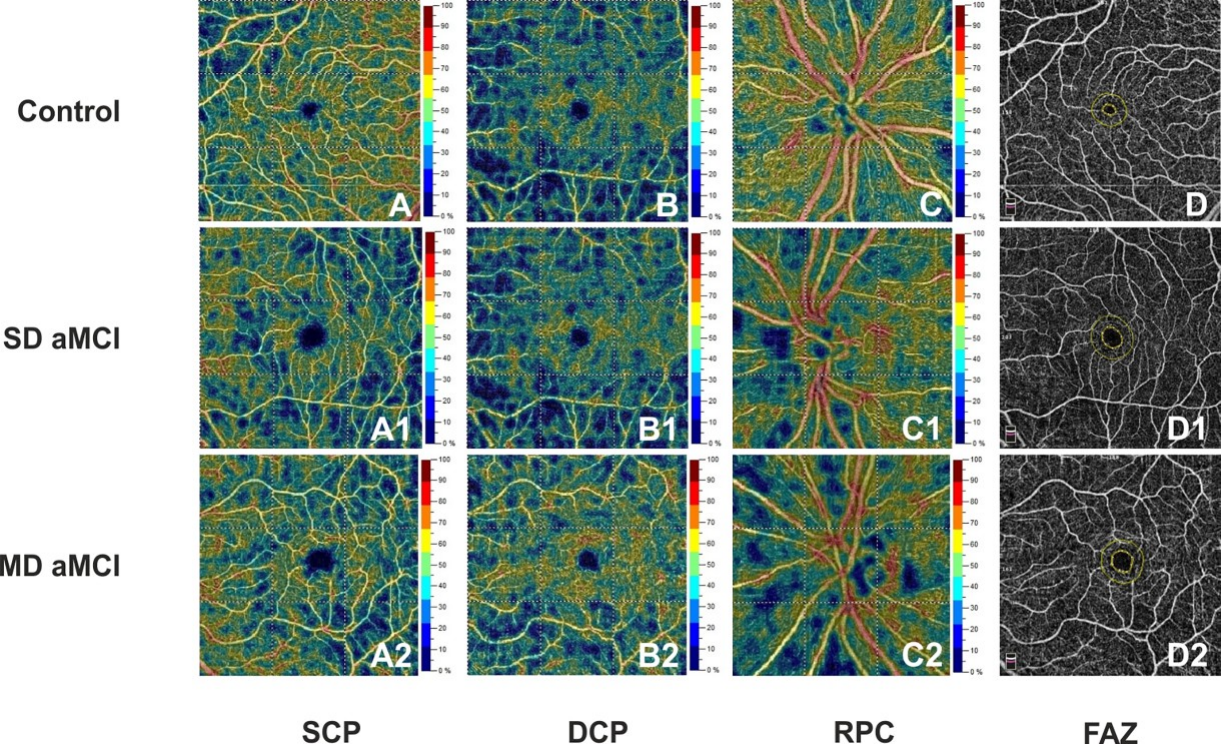
Optical coherence tomography angiography (OCTA) images of the retinal microvascular network and the foveal avascular zone in a control subject (male, 72 years) (A, B, C, D), in a patient with Single Domain amnestic Mild Cognitive Impairment (SD aMCI) (male, 70 years) (A1, B1, C1, D1) and in a patient with Multiple Domain aMCI (male, 73 years) (MD aMCI) (A2, B2, C2, D2). Compared with healthy control, the SD aMCI patient showed no differences in vessel density of superficial capillary plexus (SCP) (A1), deep capillary plexus (DCP) (B1), radial peripapillary capillary (RPC) (C1) and a significant enlargement of foveal avascular zone (FAZ) area (D1). A statistically significant reduction in vessel density of the SCP, DCP, RPC (A2, B2, C2) and a significant enlargement of FAZ area (D2) were found in the MD aMCI patient.

When comparing the two aMCI groups, VD was significantly lower in SCP in MD aMCI group (β = -4.245, p<0.001), but not in DCP, RCP and FAZ area (Fig 2).

Finally, we did not find an association between all OCTA parameters and MMSE scores in aMCI patients after correction for age, gender and disease duration. Similarly, also OCT measures did not correlate with MMSE. We reported a correlation between logic memory score and VD in the SCP (β = -0.486, p = 0.002), long term spatial memory and VD in the RCP (β = 0.577; p = 0.004), phonological verbal fluency and VD in both SCP and RCP (β = - 0.354, p = 0.034 and β = 0.413, p = 0.006, respectively); all of them did not survive after Bonferroni correction.

## Discussion

Before the onset of AD, individuals may present with MCI, defined by mild impairment on neurocognitive testing that does not affect activities of daily living [2]. Therefore, SD aMCI, MD aMCI and mild AD could be considered different clinical phenotypes that represent three severity steps along the continuum between normal aging and AD; thus representing an important target population for early intervention and disease surveillance.

Considering that cerebral and retinal vasculature show similar features, a promising avenue for biomarkers in neurodegenerative diseases is the retinal vascular network, which can be evaluated by OCTA [5] Previous studies have found decreased parafoveal VD and flow at OCTA in patients with more advanced AD, especially in rigorous studies that accounted for potential age-related changes [7,9] According to the vascular hypothesis of AD, early hypoperfusion in AD may lead to decreased Aβ clearance and subsequent plaque accumulation [31,32] and thus, vascular alterations could be early manifestations in the course of the disease.

Most of the previous OCTA investigations were performed in AD and seldom included MCI patients [6,9]. Results on MCI were conflicting and inconclusive [7,10,33]. Moreover, data on the type and number of affected domains were very poor.

Zhang et al. focused on aMCI population and found a significant decrease in parafoveal SCP VD and flow [33]. However, they did not classify the aMCI patients according to the numbers of affected domain.

Our findings showed a reduced retinal vascular network in aMCI patients compared to controls, confirming the role of vascular pathology in earlier stages of AD. In addition, when aMCI patients were divided in SD and MD, we found that the FAZ was the only OCTA parameter impaired in the SD aMCI group vs controls, while in the MD aMCI patients, beside FAZ enlargement, the VD in macular and peripapillary regions was significantly reduced. Comparing the two aMCI groups, VD was significantly lower in SCP, but not in DCP, RPC and FAZ area in MD aMCI, suggesting that the FAZ is the primary site of disease pathology, followed by the retinal vascular networks in macular and peripapillary regions.

Moreover, the FAZ enlargement seems to be the most sensitive vascular affected alteration since it has already occurred when only one domain is affected.

Considering that FAZ correlates with age and sex our data has been corrected for these aspects, to avoid age and gender related biases [34].

The FAZ area is surrounded by interconnected fine capillaries at the margin of the fovea coming from superficial and deep retinal vascular networks. This area is extremely vulnerable to hypoperfusion damages that cause the drop out of these terminal capillaries resulting in an enlargement of the FAZ [35]. Therefore, the changes in FAZ reflect the impairment of the retinal vessel density and for this reason in early stage of microvascular damages, such as in diabetic retinopathy and retinal vascular occlusions, the parameter mainly compromised is represented by the FAZ [36,37].

A FAZ enlargement was already reported in two studies on MCI and AD patients [7,9].

However, our data are in contrast with Yoon et al. and Zhang et al. [10,33], where the FAZ area was not significantly different among MCI, AD and controls.

The FAZ has been measured also in preclinical AD with variable results: unremarkable [38], or increased size with respect to healthy subjects [6].

The discrepancy among all these studies may be due to many potential confounders including the different cohort of patients and different image processing. Image processing is a crucial step to generate comparable and reliable quantitative data from retinal images but each OCTA device has a different OCTA system and a different algorithm [39]. Moreover, another essential aspect for the calculation of VD from OCTA images, is the definition of a threshold for image binarization. The most common solutions are manual binarization methods or automated binarization methods using open source software or commercial software [40,41].

Therefore, in particular, Yoon et al. used OMAG algorithm, while Zhang et al. despite using SSADA algorithm as we did, have binarized and skeletonized OCTA images using an open source thresholding algorithm [10,37]. The size of the FAZ varies even in healthy eyes of the different control groups of the studies supporting the hypothesis of a lack of standardization and homogeneity among the various image processing methods and imaging modalities [34,42].

Finally, another explanation of the contradictory data may be the different cohort of patients in the above-mentioned studies: age, race, gender and cardiovascular diseases, ocular characteristics may also affect the FAZ area, representing potential confounders [43,44].

If we consider MD aMCI as a later stage of disease compared to SD aMCI, it could be speculated that impairment of the retinal microvasculature may accelerate disease progression and mirror the spreading of the disease as evidenced by the involvement of multiple domains.

The reduced VD of the SCP in the macular region in MD aMCI patients may emphasize the significant role of the vascularization on the retinal neurodegenerative process. Indeed, the superficial retinal vascular network is located in the ganglion cell layer and is responsible for their metabolic demand [45].

A similar SCP impaired is evident also in glaucoma, another important neurodegenerative disease, that is characterized by a progressive loss of retinal ganglion cells, mainly localized in macular region [46]. Interestingly in preperimetric glaucoma (early stage of the disease) the reduction of VD in SCP precedes the structural damage of the nerve fibers [47].

Our findings support the hypothesis that microvascular dysfunction can accompany or even precede neural loss [48]. This last observation could be supported by the impairment of retinal VD in our patients in the absence of a significant vascular background, indeed we have explicitly excluded patients with cardiovascular disease, trauma, diabetes and stroke.

Macular retinal VD was reported higher in individuals with preclinical AD respect to controls [38]. Van de Kreeke, suggested that in a preclinical stage of AD the amyloid accumulation determines a retinal inflammation reaction with hypoxia and increased retinal blood flow that result in increased retinal vessel density on OCTA [38]. During disease progression, the impairment of Aβ clearance with vasoconstrictive, anti-angiogenic effects and amyloid angiopathy [14,49–51] may, cause vascular damages resulting in a progressive decrease in VD [7,9].

Assuming that the AD starts from the preclinical phase to reach the advanced phase of dementia, passing through SD and then MD aMCI, we should observe a progressive reduction of the VD in the different stages of disease, as shown in our study.

We, indeed, found no significant differences in VD in SD aMCI, and a reduced VD in MD aMCI vs CN controls. We might speculate that preclinical individuals show an increased VD induced by the initial inflammatory state. When the vascular damage evolves, we observe a progressive loss of microvasculature: initially the VD decreases to a normal level (no differences with healthy controls) that parallels the progression to SD aMCI. Subsequently a reduction in VD became evident compared to controls when the cognitive decline involves more domains. Therefore, VD in the retina may have a potential role in tracking cognitive decline, a hypothesis that needs to be validated in longitudinal studies.

MMSE has been reported to not be sensitive enough to differentiate MCI from AD likely because many factors influence cognitive tests [52,53]. This may also explain why we did not find any correlations between the MMSE score and retinal microvascular alterations in patients with aMCI.

Another explanation for the lack of correlation might rely on the not continuous distribution of MMSE scores, with most of patients belonging to the 24–26 score group.

The strength of our study is the selection of a homogenous aMCI population. To our knowledge this is one of the fist studies that focuses on aMCI patients and the first that classify them according to the number of involved domains.

The biggest limitation of our study is the small sample size that can be in part attributed the strict sampling criteria. Many elderly patients were excluded because of concomitant diseases, such as macular disease and glaucomatous optic neuropathy, which are more prevalent in this age range.

We also excluded patients with systemic diseases and history of acute myocardial infarction and stroke, all of which are prevalent in elderly patients. The low number of participants warrants caution in interpreting these results, as the results found in this subset of participants might not be applicable to the population as a whole. Moreover, we did not correlate retinal microvascular alterations with neuroimaging features of MCI. Lastly, due to the cross-sectional design of our study, we can only show a correlation but cannot establish causation between vascular changes and cognitive pathology, or exclude that our findings are an early sign of future vascular disease in our patients.

In conclusion, we suggest that FAZ may be the first changed microvascular parameter and is evident even when a single cognitive domain is impaired in aMCI patients.

Moreover, retinal vascular damages, such as the reduction of VD in SCP, become more evident later in the course of the disease, when more than one domain is involved.

Therefore, vascular network abnormalities are related to the number of domains affected and not to MMSE score.

The results of this study could help identify a subgroup within the mildly cognitively impaired population, that is at higher risk for progression to AD, who could be ideal candidates for early therapeutic intervention. OCTA could be a useful non invasive marker for early detection of vascular alterations in AD. Future studies with larger samples size and longitudinal design are needed to better elucidate whether this angiographic biomarker has sufficient sensitivity and specificity to screen for cognitive impairment, monitor disease progression, or predict future cognitive decline.
